# Quantitative assessment of the usability of electromechanical human-based modelling and simulation to replace Langendorff isolated rabbit heart experiments in the preclinical setting

**DOI:** 10.3389/fphar.2025.1671199

**Published:** 2025-10-24

**Authors:** Maxx Holmes, Hector Martinez-Navarro, Marcel Mohr, Jean-Marie Chambard, Veronique Ballet, Eva Vermersch, Ambroise Garry, Friedemann Schmidt, Blanca Rodriguez

**Affiliations:** ^1^ Department of Computer Science, University of Oxford, Oxford, United Kingdom; ^2^ R&D Preclinical Safety, Sanofi, Frankfurt, Germany; ^3^ R&D Preclinical Safety, Investigative Toxicology, Sanofi, Vitry-Sur-Seine, France

**Keywords:** modelling and simulation, new approach methodologies, cardiac electromechanics contractility, drug modelling, cardiotoxicity, cardiac risk assessment, digital twin

## Abstract

**Introduction:**

Effective proarrhythmic and inotropic risk assessment is essential for pharmaceutical development, but current preclinical methods for assessment of cardiac inotropy are flawed and costly, particularly when combined with QTc prolongation studies. *Ex vivo* rabbit Langendorff isolated heart experiments provide valuable insights into cardiovascular effects and safety, but their high cost, experimental difficulty, and limited applicability to human physiology pose challenges. Human mechanistic *in silico* modelling and simulation has proven successful in risk assessments of both electrophysiological and cardiac inotropy assessment.

**Methods:**

This study evaluates the feasibility of replacing *ex vivo* Langendorff experiments for contractility with human-based ventricular electromechanical modelling and simulations, based on 37 compounds.

**Results:**

Results show 1) 86% of compounds show qualitative agreement using four channel data (I_Kr_, I_CaL_, I_Na_, I_to_), with 73% showing quantitative agreement correlating with higher quality data, 2) sensitivity analysis identified hNCX1 and late hNaV1.5 currents as additional targets, which, when considered alongside the four channel data as input, improved agreement from 86% to 95% (at least qualitatively), 3) incomplete dose-response input data was the key reason for discrepancies between experiment and simulation, while noting only two compounds showed a complete disagreement. Incorporating patient variability through a population of N = 166 human ventricular cell models add further confidence, and highlights increasing inter-subject diversity with increasing concentrations.

**Conclusion:**

This study supports the adoption of *in silico* new approach methodologies for accurate prediction of drug cardiotoxicity, and to refine, reduce and replace the use of *ex vivo* rabbit experiments.

## 1 Introduction

The recent emergence of New Approach Methodologies (NAMs), such as *in silico* models and advanced *in vitro* systems, are bridging preclinical and clinical biomedical research by offering higher throughput, better translatability to human patients, and alleviating ethical concerns (Robertson et al., 2025). *In silico* modelling refers to computational models grounded in mathematical representations of biological systems, informed by experimental data and patho-physiological knowledge. Simulations using these tools enable prediction and mechanistic exploration of physiological and pharmacological outcomes (Viceconti et al., 2021). Advances in human modelling and simulation have enabled *in silico* drug trials as powerful tools for predicting pro-arrhythmic risk (Mohr et al., 2022; Passini et al., 2017; Trovato et al., 2022; Varshneya et al., 2021) and more recently, inotropy assessments (Trovato et al., 2025). Human-based NAMs incorporating experimental-simulation approaches enable population variability studies and simultaneous evaluation of proarrhythmic and inotropic effects. Regulatory bodies like the United States Food and Drug Administration (FDA) and European Medicines Agency (EMA) endorse *in silico* methods for preclinical proarrhythmic risk assessments (EMA, 2022; Musuamba et al., 2021; Valentin and Leishman, 2023). However, NAM adoption in regulatory evaluations remains limited, highlighting the need for robust evaluation processes and better co-ordination (Valentin and Leishman, 2025).

Animal models (*in vitro*, *in vivo* and *ex vivo*) are widely used to evaluate proarrhythmic cardiotoxicity, using metrics like drug-induced repolarisation prolongation as surrogates for QT prolongation in the whole heart (Gintant et al., 2016). While current guidelines focus on QT prolongation and proarrhythmic risk, broader assessments of cardiotoxicity including inotropic risk are essential to improve safety assessments. Excessive inotropic cardiotoxicity has led to drug discontinuation (Mamoshina et al., 2021), yet inotropic evaluations lack equivalent rigour, potentially halting valuable compounds prematurely. Preclinical assessment of drug-induced cardiac inotropy remains challenging, as many animal-based or human induced pluripotent stem cell (iPSC)-derived cardiomyocyte models fail to capture human primary cardiomyocyte physiology (van Meer et al., 2016), or lack mature inotropic mechanisms (Pointon et al., 2015).

The Langendorff rabbit isolated heart model is widely used in cardiovascular research, enabling studies of the heart’s intrinsic properties in isolation of the body’s other dynamic systems. It facilitates investigation of contractile function, electrical activity and coronary blood flow in isolation which makes it valuable for pharmaceutical drug testing (Roche et al., 2010). However, in addition to ethical limitations, limited viability of isolated hearts mean experiments are time-sensitive, and species differences limit the translation of experimental outcomes to clinical risk assessments (Blanset et al., 2020; Van Norman, 2020).

The goal of this study is to quantitatively assess the usability, and optimise the translatability, of *in silico* simulations using human ventricular cardiomyocytes electromechanical models to replace *ex vivo* isolated rabbit experiments. Simulations for 37 compounds, containing both proprietary and reference compounds, are benchmarked with proprietary data from previously conducted rabbit Langendorff isolated heart experiments. Simulations incorporate variations in ionic conductance across the human population to support the development of “Phase 0” *in silico* cardiovascular studies, considering different patient populations. Sensitivity analysis identifies relevant currents beyond the four ion channel assay panel used in Comprehensive *in vitro* Proarrhythmic Assay (CiPA)-based studies (Colatsky et al., 2016) and identified by Zhou et al. (2020) as the minimum set required for pro-arrhythmia predictions. We quantify the importance of integrating high quality experimental data, which is crucial for *in silico* predictivity, and we pave the way towards the replacement of animal-based *ex vivo* experimentation.

## 2 Materials and methods

**FIGURE 1:**
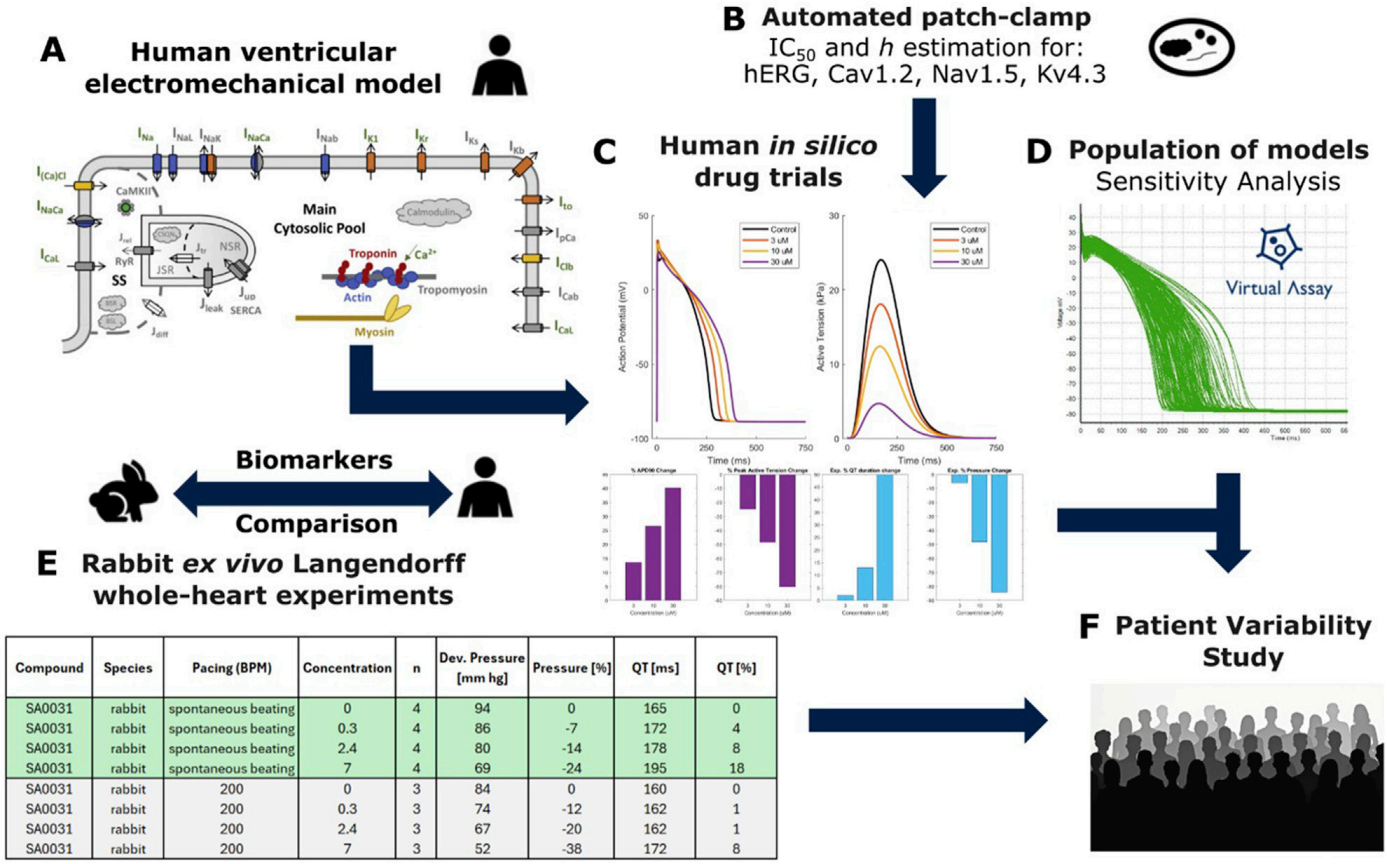
Combined experiment-simulation pipeline used to perform this study. **(A)** Structure of the biophysically detailed computational model used to simulate human ventricular cellular electromechanics (Margara et al., 2021). **(B)** *In vitro* estimation of IC50s and Hill coefficients through automated patch clamp. **(C)** Single-cell *in silico* drug trials performed using simple pore-block models, output traces showing action potential (AP) and active tension with comparison to rabbit experimental data. **(D)** Experimentally calibrated population of 166 models generated using the Virtual Assay Software (Oxford University Innovation ^©^ 2018); green traces show simulated AP. **(E)** *In silico* results were compared against pre-existing *ex vivo* recordings from whole-heart Langendorff rabbit experiments, **(F)** Drug models and calibrated population of models used to investigate patient variability in contractility for select compounds.

Figure 1 outlines the study’s methodology. A diverse selection of 37 unique compounds was analysed to measure drug-induced changes in both repolarisation and contractility properties *in vitro* in the isolated rabbit heart and *in silico* in human electromechanical cardiomyocyte simulations. These data were compared to evaluate and optimise the potential of human-based electromechanical simulations as alternatives to animal experimentation.

We employed the human-based electromechanical ventricular cardiomyocyte model (ToR-ORd-Land, Figure 1A), featuring biophysically detailed models of electrophysiology, excitation-contraction coupling and contractility mechanisms including ionic current, calcium dynamics and cross-bridge formation. This was performed through coupling of the ToR-ORd model (Tomek et al., 2019) with the Land et al. contractility model (Land et al., 2017) as in Margara et al. (2021), with additional studies also demonstrating validation through comparison with experimental data (Trovato et al., 2025). The choice of this model was based on extensive validation conducted through comparison with experimental data in control conditions and following pharmacological interventions and disease conditions (Tomek et al., 2019; Zhou et al., 2024b; Zhou et al., 2024a). Input data to the model included the automated patch-clamp quantifying the half-maximal inhibitory concentrations (IC50s) for four cardiac ion channels for each compound (Figure 1B).

Simulations yielded action potential, calcium, and active tension (AT) transients in single cells, in addition to all underlying variables such as ionic currents to mechanistically explore drug effects (Figure 1C). A sensitivity analysis, using Virtual Assay software (Passini et al., 2021), was performed using a population of human ventricular models (N = 166) to identify key determinants of repolarisation and contractility changes in healthy populations (Figure 1D). *In silico* predictions of action potential duration (APD) and peak active tension were compared with experimental rabbit whole-organ QT and pressure measurements (Figure 1E). Cardiac cell models further assessed patient variability in repolarization and contractility for selected compounds (Figure 1F). Single cell simulations were chosen in this study to minimize their computational cost (compared to whole ventricular simulations) and enable substantial exploration of compounds and patient variability.

### 2.1 Experimental data

#### 2.1.1 *In vitro* ion channel data

Following the procedure in Trovato et al. (2022), we used automated patch-clamp platforms to study four ionic currents relevant to human repolarisation, as outlined by Zhou et al. (2020) as the minimum set of ion channels, marked by the CiPA Ion Channel Work Group, for reliable predictions of repolarisation abnormalities. These channels are the human ether-a-go-go related gene potassium channel also known as hERG channel, modulating the rapid inward rectifying potassium current; hCav1.2, modulating L-type calcium current; hNav1.5, modulating peak sodium current; and hKv4.3, modulating the transient outward potassium current. hKv4.3 notably has significant inter-species differences between human and rabbit (Oudit et al., 2001). The QPatch system (Sophion, Denmark) was used to investigate hERG, hNav1.5 and hKv4.3, and the SyncroPatch system (Nanion, Germany) was employed for hCav1.2. Recordings for all 37 compounds were performed at 21 °C.

Furthermore, *in vitro* potency for 11 compounds was assessed on the NCX isoforms using a cell-based calcium mobilization assay on CHO cell lines expressing NCX1, NCX2 or NCX3. Measurements of intracellular calcium concentration employed a fluorescent imaging plate reader (Molecular Devices, United States) with the calcium-sensitive dye Fluo4-AM. Detailed methods are described in Pelat et al. (2021).

IC50 data for all compounds are summarised in Supplementary Table S1. When a Hill coefficient was not available, it was approximated as 1. Compounds that did not inhibit a channel within the tested concentrations were interpreted as having no effect and are given no value. This is relevant for Kv4.3, in which more than half of the compounds showed no effect. Some compounds showed a visible inhibition, but an IC50 was not achieved. These compounds were taken at their maximum possible concentration, or with sufficient data, an IC50 was estimated with a Hill coefficient of 1.

#### 2.1.2 *Ex vivo* rabbit whole-heart Langendorff experiments

Routine experimental measurements used in this study were retrieved from already existing datasets collected and curated by Sanofi, contributing to the 3R principles in animal experimentation: replacement, reduction and refinement. All the procedures described in the present study were performed in agreement with the European regulation (2010/63/EU) and under the approval and control of Sanofi’s ethics committee. All procedures were performed in Association for Assessment and Accreditation of Laboratory Animal Care International (AAALAC)-accredited facilities, in full compliance with the standards for the care and use of laboratory animals and in accordance with the French Ministry for Research. Hearts were excised from anesthetized rabbits (Medotomidine (Domitor^®^): 150 μg/kg, Ketamine (Imalgene 1,000^®^): 10 mg/kg), washed in heparinized physiological solution at room temperature, and perfused through the aorta using the Langendorff method (EMKA-Technologies, France). The physiological solution (in mmol/L: NaCl 120; KCl 4; MgCl2 1; NaH2PO4 1.8; NaHCO3 25; glucose 11; CaCl2 1.8; pH = 7.4) was maintained at 37 °C ± 0.5 °C, gassed with 95% O2/5% CO2 and delivered into the heart through a cannula inserted into the ascending aorta. Retrograde flow in the aorta closed the leaflets of the aortic valve, and consequently, the entire perfusate entered the coronary arteries via the ostia at the aortic root. After passing through the coronary circulation, the perfusate drained into the right atrium via the coronary sinus. The ventricles were empty of perfusate throughout the experiment. The experimental chamber was closed to maintain a wet atmosphere.

Hearts were spontaneously beating. Left ventricular contractility was derived from the developed left ventricular pressure using both developed pressure and maximal rate of rise. The left ventricular pressure was evaluated using a pressure transducer within a saline-filled latex balloon inserted into the ventricular chamber. The saline volume allowed a resting pressure of 10–20 mmHg (preload). The electrocardiogram was recorded with two flexible electrodes gently pressed on the epicardium. The ECG parameters PQ, QRS, and QT durations were calculated using specific software (HEM-Notocord systems).

After stabilization, pressure was recorded with the vehicle for 15 min and used as a control period. Then, compounds were consecutively perfused at four concentrations (C1, C2, C3, and C4) during four successive periods of 10 min, followed by a 30-min washout period after the last concentration.

### 2.2 Simulating human cellular electrophysiology and contractility

Human ventricular electrophysiology, calcium dynamics and active contraction were simulated with the ToR-ORd model (Tomek et al., 2019) coupled with the Land contractility model (Land et al., 2017). The ToR-ORd model comprises of 17 ionic currents and fluxes, and has been validated under healthy, disease and drug conditions with extensive experimental data. It can produce crucial arrhythmia mechanisms at the cell level, including early after-depolarisations, an established, mechanistically-sound metric to quantify pro-arrhythmic cardiotoxicity *in silico* (Passini et al., 2021). As described in Margara et al. (2021), the ToR-ORd model is bidirectionally coupled to the Land model through the free intracellular calcium concentration. This is computed in the ToR-ORd model and used as input in the Land model to generate active tension, while the amount of calcium bound to troponin C is fed back to the ToR-ORd model and used to update the free intracellular calcium concentration at each time step.

A virtual population of 500 human ventricular endocardial cellular electromechanical models based on the ToR-ORd-Land model was generated by scaling ionic current conductivities (50%–150%) using Latin Hypercube Sampling. After 500 stimuli (1 Hz), 166 models meeting experimentally obtained criteria for human ventricular action potential morphology, calcium transient and tension biomarkers (Table 1) were retained for simulations (Figure 1D).

**TABLE 1:** Calibration criteria and calibrated biomarkers for the population of healthy adult cardiomyocyte models. Calibration criteria for healthy adult ventricular myocytes and simulated biomarker outputs from the calibrated virtual population. Action potential morphology, calcium transient, electromechanical, and contractile biomarkers in control for the ToR-ORd-Land model (Margara et al., 2021). Mean and standard deviations calculated from the virtual population of 166 cell models are provided. The baseline values are similar or identical to the population mean for all biomarkers.

Marker		Name	Unit	Calibration range		Virtual population	
				Min value	Max value	ToR-ORd-Land	Population
Action potential morphology	APD40	Action potential duration (40% recovery)	ms	85	320	195	189 ± 34
	APD50	Action potential duration (50% recovery)	ms	110	350	220	214 ± 40
	APD90	Action potential duration (90% recovery)	ms	180	440	273	267 ± 46
	APD95	Action potential duration (95% recovery)	ms	180	500	280	274 ± 47
	Tri90-40	Action potential triangulation (APD90 - APD40)	ms	50	150	78	77 ± 19
	max (dV/dt)	Maximum upstroke velocity	mV/ms	100	1,000	347	340 ± 66
	Vmax	Peak voltage	mV/ms	10	55	33	32 ± 6
	RMP	Resting membrane potential	mV/ms	−95	80	−89	−88.5 ± 0.4
	qNet	Net charge	uC/uF	−1	1	0	−0.02 ± 0.06
Calcium transient	CTD50	Calcium transient decay (50% recovery)	ms	120	420	201	203 ± 33
	CTD90	Calcium transient decay (90% recovery)	ms	220	785	352	365 ± 54
	CTD95	Calcium transient decay (95% recovery)	ms	220	1,000	402	418 ± 62
	CaTmax	Maximum intracellular calcium concentration	mM	200	1,000	466	505 ± 78
	CaTamp	Calcium transient amplitude	mM	200	600	394	431 ± 73
	CaiD	Intracellular diastolic calcium concentration	mM	0	400	72	74 ± 7
Electromechanics and contractility	EMw90	Electromechanical window (ATttp + ATrt90 - APD90)	ms	0	1,000	79	98 ± 65
	EMw95	Electromechanical window (ATttp + ATrt95 - APD95)	ms	0	1,000	122	144 ± 73
	ATpeak	Active tension peak	kPa	15	40	23	27 ± 7
	ATttp	Active tension time to peak	ms	120	200	168	173 ± 14
	ATrt50	Active tension decay time (50% recovery)	ms	90	140	114	117 ± 10
	ATrt90	Active tension decay time (90% recovery)	ms	200	600	233	229 ± 16
	ATrt95	Active tension decay time (95% recovery)	ms	200	600	266	272 ± 18
	dATmax	Maximum active tension gradient	kPa/ms	0	10	0	0.29 ± 0.07
	dATmin	Minimum active tension gradient	kPa/ms	−10	0	0	−0.16 ± 0.04

### 2.3 Human *in silico* drug trials

**FIGURE 2:**
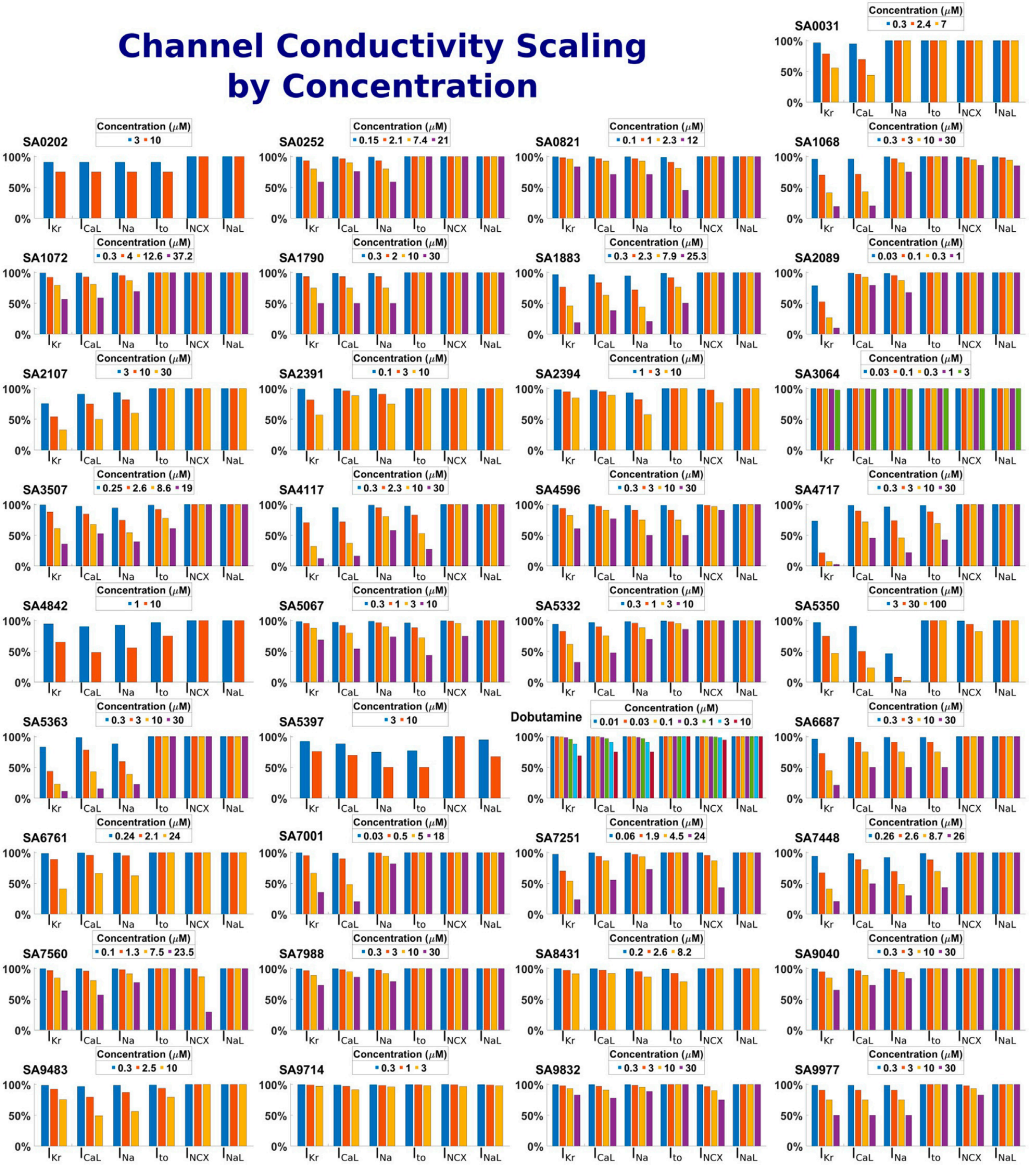
Summary of drug-induced effects on six cardiac ion channels. Channel modulation was calculated using a simple pore-block model. Each panel shows one of the 37 compounds, both reference and proprietary, with different bars representing the residual current as a percentage following drug-application for each ion channel and drug concentration.

Drug-induced inhibition of ion channel function was simulated through conductance scaling using a simple pore-block model (Brennan et al., 2009), with the experimental IC50, hill coefficients and drug concentrations reported in Supplementary Table S1 for the minimum set of ion channels required for reliable predictions of repolarisation abnormalities set out by Zhou et al. (2020): I_Kr_, I_CaL_, I_Na_, I_to_ and additionally I_NCX_ and I_NaL_ which were determined as important through a sensitivity analysis. Figure 2 shows a visual representation of the conductivities of these currents following the application of the drug model for each compound at each concentration.

Starting from the steady state described above, the models were paced for 200 beats at 1 Hz using a stimulus current, with AP, intracellular calcium transient (CaT) and active tension biomarkers computed using the last beat. The final 10 beats were checked for repolarisation abnormalities, defined as the existence of a positive change in membrane potential with respect to time greater than 0.01 mV/ms occurring after the first 100 ms of the beat. If a repolarisation abnormality was observed, the biomarkers were calculated for the last beat in normal sinus rhythm. Drugs were initially simulated using the minimum four ion channels needed as detailed in Section 2.1.1, and then the 12 compounds for which hNCX1 and late hNav1.5 inhibitions were experimentally measured were simulated again including these additional drug/ionic current interactions.

All simulations were performed on a regular laptop (Intel Core i7-1185G7, 16 GB RAM). The time required to simulate one drug at one concentration (500 beats at 1 Hz) on all 166 cell models was 14 min.

### 2.4 Metrics for comparison of experiments and simulations

Baseline biomarker measurements were established in control conditions for both *ex vivo* experiments and *in silico* simulations, benchmarking compound results at each concentration against these respective controls. Percent variations of means for experiments and simulations were calculated. In the population, these variations were measured against each cell’s control, and the group mean is calculated.

For repolarisation assessment, *in silico* APD_90_ prolongation was compared to experimental QT prolongation *ex vivo*, as these biomarkers are both related to ventricular repolarisation (Ducroq et al., 2007; Omata et al., 2005; Redfern et al., 2003; Romero et al., 2018). Contractile assessment compared *in silico* peak active tension with *ex vivo* pressure. Increased ventricular pressure results from myocardial contraction-generated tension, measured at the left ventricular wall. The measured pressure is the result of the collective forces of active (contractility) and passive tension (preload). These factors–active contractility, passive tension, blood volume, heart rate and arterial pressure–all influence pressure, though arterial pressure is less relevant in Langendorff heart experiments with retrograde fixed perfusion at 60 mmHg.

To evaluate consistency between experimental outcomes and human *in silico* trial predictions, we used a quantitative metric based on the percentage difference between experimental QT prolongation and simulated APD prolongation, as well as experimental contractility and simulated active tension under drug influence at each concentration. Three outcomes were defined: 1) quantitative agreement, for matching trends with ≤25% difference (increase, decrease or negligible response, where negligible was defined as a change of no more than ±10% from control at every concentration tested); 2) qualitative agreement, for matching trends but >25% difference; and 3) disagreement, for differing trends, excluding negligible differences (e.g., negligible increases vs. decreases). A compound’s match was determined by the majority of concentrations tested. For some compounds, the highest concentration produced a severe change in active tension or APD, inconsistent with the general trend, and caused by general depolarisation/contraction failure. In these cases, we excluded the highest concentration from the analysis, as it was not representative of the general trend.

Repolarisation and contractility assessments were analysed separately. Compounds were classified as full matches (both assessments in qualitative or quantitative agreement), partial matches (one match in disagreement) or mismatches (both in disagreement).

### 2.5 Verification, validation and uncertainty quantification strategy for credibility assessment

This study aims to assess and optimise a translational model linking *ex vivo* Langendorff rabbit heart experiments and human-based *in silico* trials. Comparing simulation outcomes with experimental data and established methods is critical for validating the computational framework’s credibility in assessing drug effects and potential cardiotoxicity.

The ToR-ORd model, coupled with the Land model, was evaluated against a wide range of experimental data in previous studies with drug action as a specific focus (Tomek et al., 2019; Margara et al., 2021). We also confirmed its consistency in the Virtual Assay software through comparison with MATLAB implementations. The computational pipeline accurately reproduced drug-induced alterations in cell markers for reference compounds in the dataset, including hydroxychloroquine, loperamide, clozapide, lidocaine and cannabidiol.

Moreover, a sensitivity analysis (SA) was conducted on ion channel conductivities of the four primary currents (I_Kr_, I_CaL_, I_Na_, I_to_) and extended to a total of 10 key currents (I_Ks_, I_K1_, I_NCX_, I_NaL_, J_rel_, J_up_) in the human ventricular cardiomyocyte. By modulating conductance from 10%–100% of the baseline value, the SA identified key mechanisms influencing contractile and repolarisation biomarkers in healthy human ventricular cell models (described in Section 2.2).

## 3 Results

### 3.1 *In silico* predictions using drug block for four ionic currents as input compared with *ex vivo* observations

**FIGURE 3:**
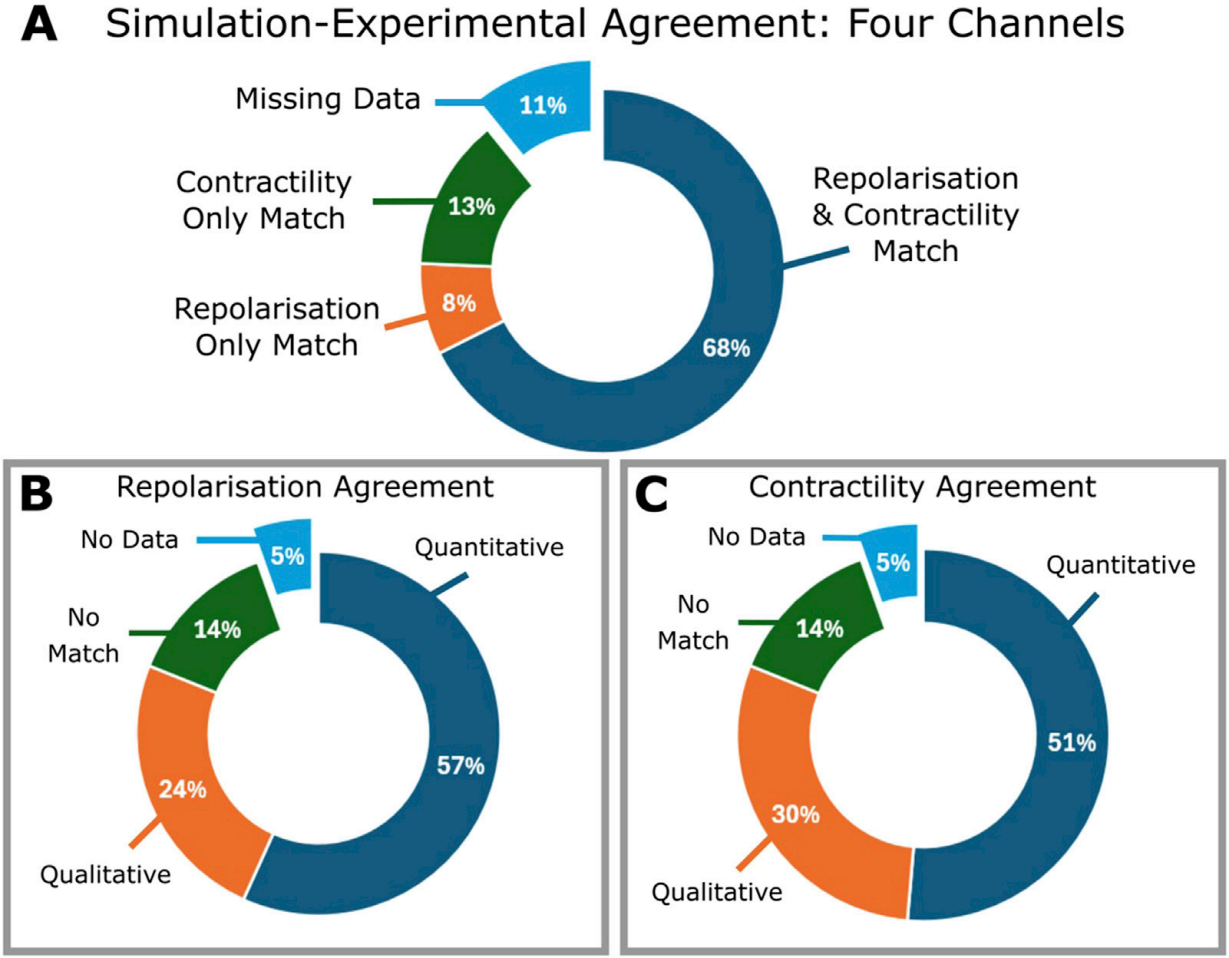
Summary of the comparison between *ex vivo* experiments and *in silico* testing based on blocking four ion currents (IKr, ICaL, INa and Ito) of all 37 compounds. Percentages are calculated including compounds for which experimental inotropic or ECG data is missing. **(A)** Pie chart showing percentages of compounds which demonstrated a match one or both of APD prolongation or contractility; for all compounds evaluated, one compound was found to exhibit no match between either APD or contractile comparisons. **(B)** Contractility match breakdown into quantitative (within 25%) or qualitative agreement, no match, or missing data. **(C)** APD match breakdown into quantitative, qualitative, no agreement or missing data.

Figure 3 summarizes the comparison of experiment and simulation results for the 37 compounds, characterised by a minimum dataset of four ion currents (I_Kr_, I_CaL_, I_Na_ and I_to_). Individual results are presented in Supplementary Table S1. All compounds with *ex vivo* data show agreement in at least either electrophysiology or contractility, and 68% of compounds show matches for both. Matches with only contractility or repolarisation agreement account for 13% and 8%, respectively. Quantitative matches were found for 73% of compounds. Of the 11% missing experimental data for either repolarisation or contractility, all but one compound exhibited at least a qualitative match.

Excluding compounds without contractility data, 86% showed agreement, of these, 54% were quantitative, 31% were qualitative and 14% (5 compounds) had no match. Similarly, excluding those without repolarisation data (Figure 3C), 86% demonstrated agreement: 60% quantitative, 26% qualitative, and 5 compounds (14%) presented no match.

**FIGURE 4:**
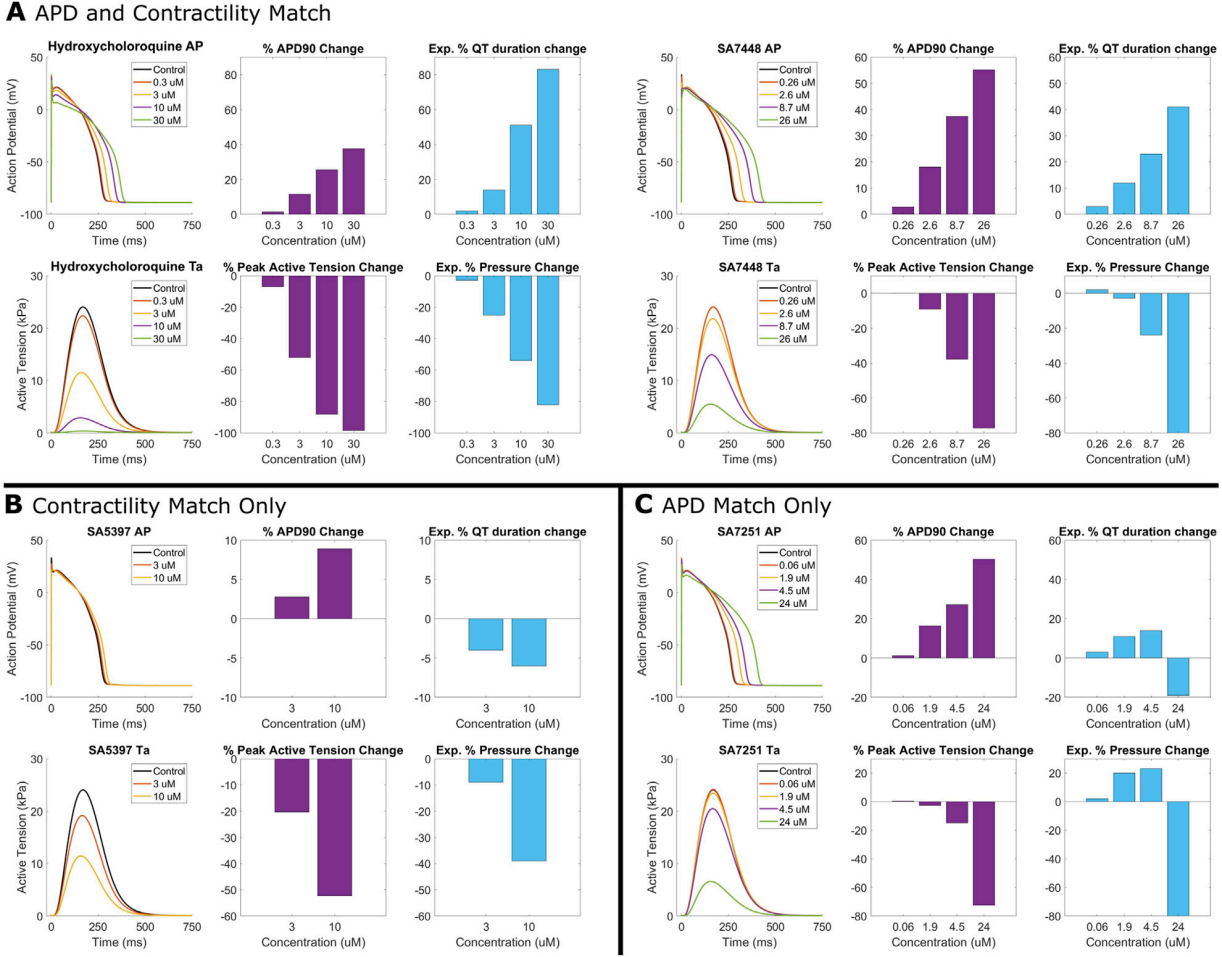
Comparison between human *in silico* and rabbit *ex vivo* biomarkers. Percentage modification of QT prolongation and pressure measurements taken from *ex vivo* Langendorff isolated rabbit heart experiments (purple) are compared with APD prolongation and active tension from simulated human ventricular myocytes (blue). **(A)** Quantitative match for both APD prolongation and contractility for hydroxychloroquine (right), and SA7448, a proprietary compound. **(B)** Quantitative agreement for contractility for SA5397 but only a qualitative agreement for APD/QT prolongation. **(C)** Quantitative agreement for APD/QT prolongation for SA7251, but opposite trend in contractile modulation.

Figure 4 illustrates four examples comparing experimental and simulated drug effects. For hydroxychloroquine (Figure 4A), simulations reveal a −58% decrease in contractile force, aligning with observed contractility loss in porcine heart slices (Wu et al., 2023) and human ventricular cardiomyocytes (Jordaan et al., 2021). A 26% APD_90_ prolongation at 10 µM quantitatively agreed with experiments in guinea-pigs (Wang et al., 2021) and engineered cardiac tissue from human stem cell cardiomyocytes (Wong et al., 2021). Additionally, Figure 4 also presents three proprietary compounds further exemplifying simulation-experimental agreement. SA7448 (Figure 4A) shows quantitative agreement for both repolarisation and contractile modulation across all doses. SA5397 (Figure 4B) reproduced contractile effects but predicted dose-dependent APD prolongation absent in *ex vivo* QT data due to negligible effects on the QT interval. SA7251 (Figure 4C) matched QTc and simulated APD, while simulated contractility contradicted experimental pressure measurements. This was linked to missing L-type calcium channel inhibition data for SA7251 (Supplementary Table S1).

**FIGURE 5:**
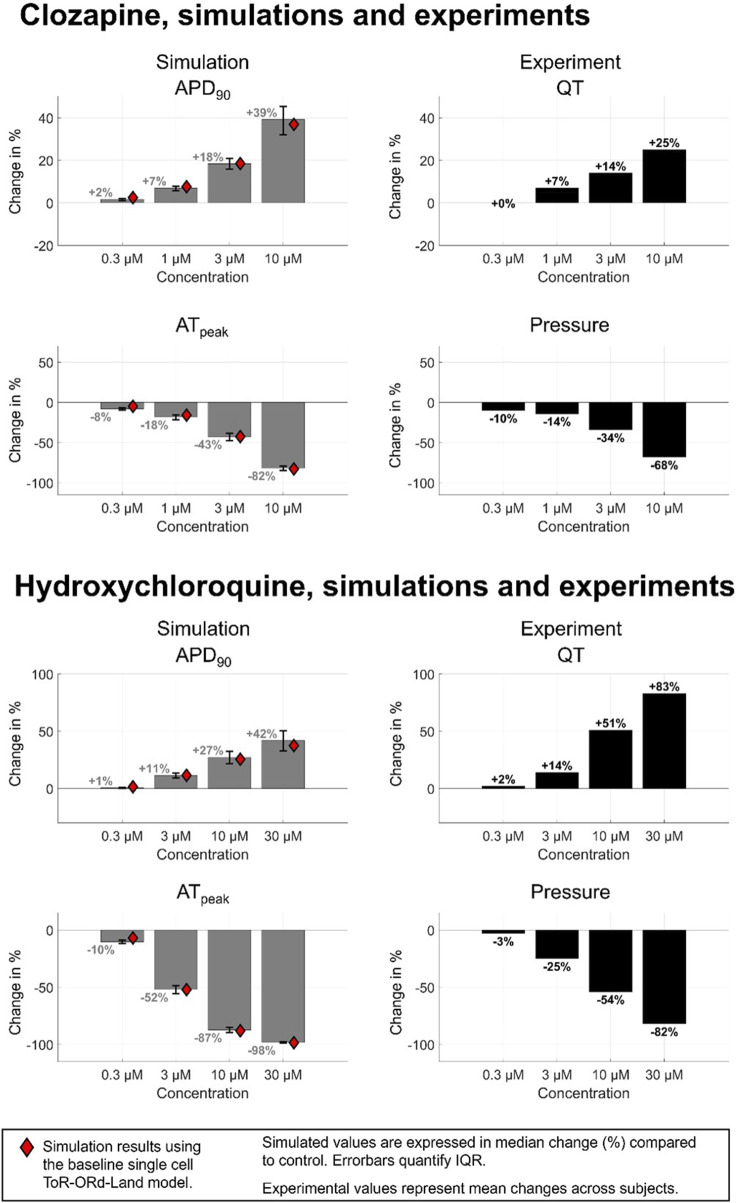
Comparison between human *in silico* and rabbit *ex vivo* biomarkers for 2 compounds presenting a quantitative match for both APD and contractility changes in single cell: clozapine and hydroxychloroquine. *In silico* markers (grey bars) quantified from a healthy-calibrated population of human ventricular cardiomyocytes (N = 155), values shown is the median, error bars show the interquartile range, and red rhombus represent the measurements obtained from single cell using the baseline ToR-ORd-Land model. The inclusion of biological variability provides further confidence in the robustness of the simulation results. IQR = Inter-quartile range.

Figure 5 demonstrates effect of two reference compounds, hydroxychloroquine and clozapine on populations of human ventricular myocytes, both showing quantitative agreement with internal experimental data from 0.3 to 10 µM, and quantitative agreement at 30 µM. APD variability increases at higher concentrations. Clozapine shows a +1.70% [IQR: (+1.14%, +2.01%)] APD prolongation at 0.3 µM, increasing to +39.17% [IQR: (+32.11%, +45.30%)] at 10 µM. Clinical data shows Clozapine produced a dose-dependent QTc prolongation, but clinically significant prolongation is rare; published studies show either minor (∼10 ms) or no QTc prolongation at indicted dosages (Grande et al., 2011; Wenzel-Seifert et al., 2011). Hydroxychloroquine simulations predict a +0.53% [IQR: (+0.08%, +0.89%)] APD increase at 0.3 µM, rising to +33.53% [IQR: (+24.48%, +41.98%)] at 30µM, with greater response variability. Simulated APD prolongation via hydroxychloroquine on QT matches with clinical data (N = 1890, 86% male; El Kadri et al., 2022) suggesting hydroxychloroquine has a low incidence of severe QTc prolongation at indicted dosages (up to approximately 1 µM), and an average 11 ms increase in QTc when administered in isolation (El Kadri et al., 2022; Morrisette et al., 2020). Cell population simulation results align with single-cell simulations (red diamonds in Figure 5), yet greater variations at higher concentrations emphasizes the need to account for population variability.

### 3.2 Optimisation of translational model through sensitivity analysis

The sensitivities of APD90, peak active tension (ATpeak), time to peak active tension (ATttp) and decay to 90% active tension (ATrt90) to the reduction of 10 ionic currents are shown in Table 2. In our analyses, APD90 has the largest dependence on I_Kr_ (+34.5% at 50% block), followed by I_CaL_, I_NaL_ and I_NCX_ where inhibition corresponded with reductions in APD90. ATpeak was strongly dependent on I_NCX_ where inhibition led to largely increased peak tension (+97.6% at 50% block) and I_CaL_ where inhibition reduced peak tension (−85.4% at 50%). ATpeak had lesser dependencies on J_rel_, J_up_, and potassium currents. ATttp and ATrt90 is mostly influenced by calcium currents (I_CaL_, J_up_).

**TABLE 2:** Sensitivity analysis of APD and AT biomarkers with respect to changes in the conductivities of the main ionic currents in the human ventricular cardiomyocytes, conducted on a N = 166 variants of the ToR-ORd-Land model. For each current, conductance was modulated to 75%, 50%, 25% and 10% of the baseline value; changes in APD and contractility markers (AT_peak_, AT_ttp_, AT_rt90_) are quantified as change (%) in comparison to baseline. The divergent color map represents the magnitude and direction of the marker change (red: increase, blue: decrease).

Ionic current	Remaining current	>50% cells depolarisation failure	APD_90_	AT_peak_	AT_ttp_	AT_rt90_
INa	75%	-	−0.4%	0.9%	1.2%	−0.5%
	50%	-	0.5%	3.9%	2.6%	−0.6%
	25%	Yes	−85.2%	−99.8%	−98.4%	−99.4%
	10%	Yes	−88.4%	−99.8%	−98.4%	−99.5%
ICaL	75%	-	−4.9%	−51.0%	−5.4%	−4.7%
	50%	-	−10.4%	−85.4%	−9.0%	−8.3%
	25%	-	−18.8%	−98.6%	−16.3%	−7.6%
	10%	-	−23.3%	−99.7%	−16.3%	7.6%
IKr	75%	-	13.5%	7.0%	2.0%	0.2%
	50%	-	34.5%	15.9%	3.5%	0.9%
	25%	-	69.3%	17.1%	3.7%	10.9%
	10%	-	105.9%	0.6%	2.3%	14.7%
INaL	75%	-	−5.9%	−10.6%	1.6%	−1.2%
	50%	-	−10.9%	−18.5%	3.2%	−3.3%
	25%	-	−15.8%	−26.4%	3.5%	−5.4%
	10%	-	−18.5%	−30.6%	5.0%	−6.9%
Ito	75%	-	−0.7%	−4.9%	0.5%	−0.5%
	50%	-	−0.3%	−8.0%	0.5%	−0.5%
	25%	-	0.2%	−11.5%	0.5%	−0.5%
	10%	-	0.4%	−13.8%	0.5%	−0.5%
IKs	75%	-	−0.9%	−2.5%	0.6%	−0.5%
	50%	-	−0.7%	−2.2%	0.6%	−0.5%
	25%	-	−0.5%	−1.9%	0.6%	−0.4%
	10%	-	−0.4%	−1.8%	0.6%	−0.3%
IK1	75%	-	1.0%	−3.2%	−0.3%	−0.7%
	50%	-	3.6%	−4.3%	0.0%	−1.0%
	25%	-	6.9%	−6.9%	−0.6%	−1.6%
	10%	Yes	2.7%	−99.8%	289.4%	−2.4%
INCX	75%	-	−7.1%	32.6%	3.6%	4.5%
	50%	-	−14.0%	97.6%	8.6%	16.9%
	25%	-	−22.8%	193.9%	18.4%	51.8%
	10%	-	−34.5%	260.4%	32.4%	99.3%
Jrel	75%	-	−1.3%	−7.4%	0.6%	−0.5%
	50%	-	−1.6%	−14.8%	1.0%	−0.3%
	25%	-	−2.3%	−27.5%	2.1%	0.0%
	10%	-	−3.1%	−42.3%	5.0%	−0.3%
Jup	75%	-	−0.1%	−19.0%	8.5%	7.4%
	50%	-	0.5%	−37.7%	20.9%	11.7%
	25%	-	1.4%	−57.8%	37.9%	26.0%
	10%	-	3.1%	−68.3%	51.9%	47.2%

The currents in the standard CiPA assay panel (I_Na_, I_CaL_, I_Kr_ and I_to_) are routinely quantified. In our analyses, I_Na_, I_CaL_ and I_Kr_ show significant effects on electrophysiology and contractile markers. Severe I_Na_ block caused depolarization failure (<25% remaining current). I_CaL_ block leads to mild APD90 reduction, but critical contractility failure. I_Kr_ block led to large APD prolongation, and mild effects on contractility. I_to_ block was only shown to mildly reduce ATpeak.

These results highlight I_NaL_ and I_NCX_ as additional currents with significant impact on our selected biomarkers. I_NaL_ block moderately reduced APD90 (−10.9% at 50% block) and ATpeak (−18.5% at 50% block). I_NCX_ block also reduced APD90 moderately (−14% at 50% block) but large ATpeak increase (+97.6% at 50% block) and a relevant impact on ATrt90 (+16.9% at 50% block). We performed further experiments exploring the impact of including I_NCX_ and I_NaL_ in 12 compounds, focused on those with lesser agreements.

### 3.3 Inclusion of hNCX1 and late hNav1.5 inhibition *in vitro* data improves agreement between simulations and experiments

**FIGURE 6:**
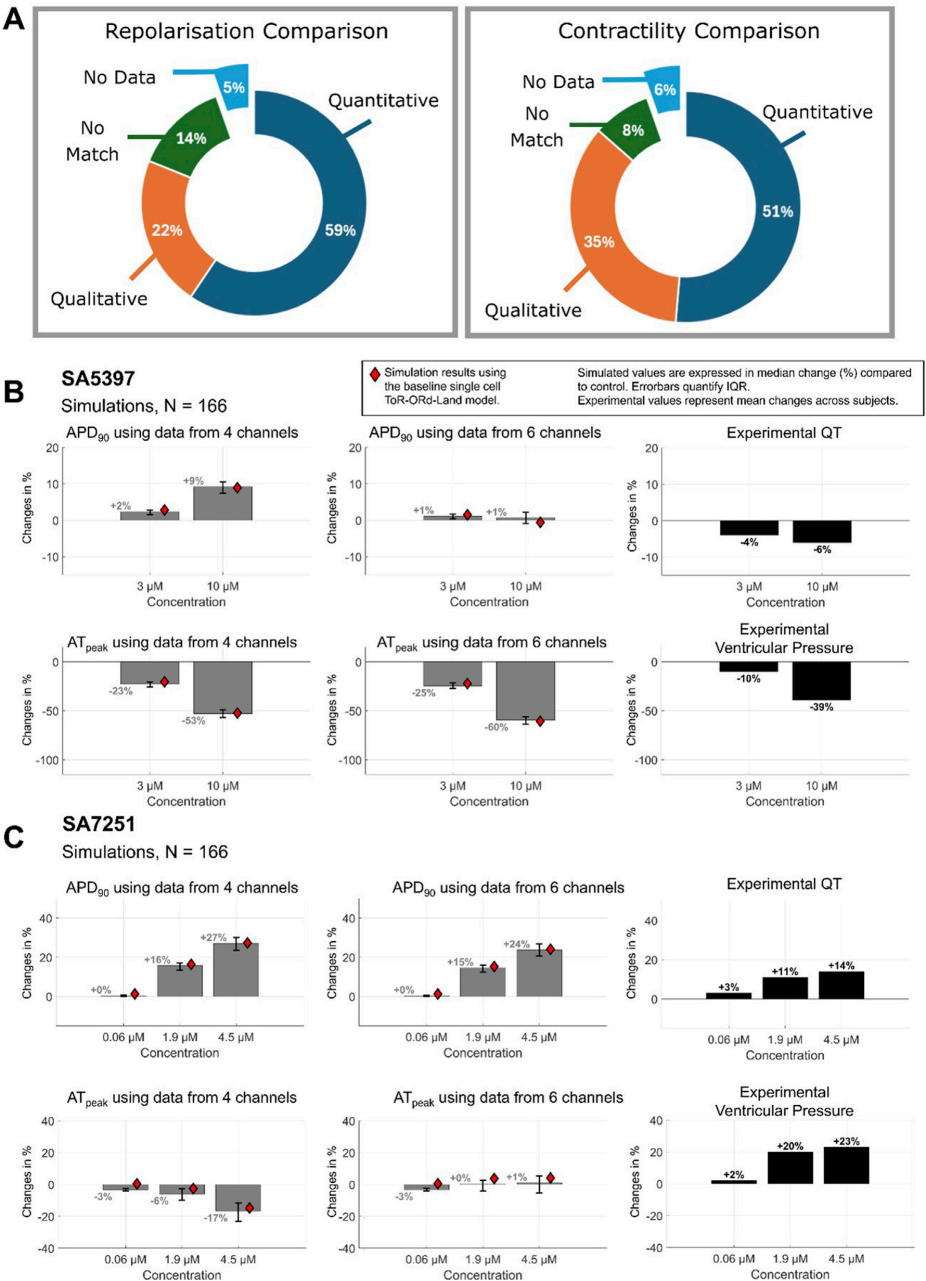
Simulation-Experimental Agreement with 6 channels. **(A)** Inclusion of NaV1.5 late sodium inhibition (late hNav1.5) and NCX1 data improves agreement for compounds **(B)** SA5397 and **(C)** SA7251, respectively. Compared to Figure 2 without this data, we observe a minor increase in contractile agreement and a major increase in APD-QT prolongation agreement.

We tested the hypothesis that consideration of I_NCX_ and I_NaL_ inhibition would improve the match between experiments and simulations. Of the 12 compounds which displayed qualitative, or no agreement based on 4 ion channel data, we found that 6 compounds notably inhibit either I_NCX_ and I_NaL_ (Supplementary Table S1). Furthermore, Figure 6A summarises simulation-experiment agreement after including I_NCX_ and I_NaL_ inhibition. Quantitative agreement between simulated APD and experimental QT improved for one compound, and contractility agreement increased for three compounds–shifting from no match to qualitative agreement. This increased the number of compounds with agreement to 95% (35 of 37).

Significant improvement was observed for four compounds (SA9714, SA7251, SA5397, SA5067) and minor improvement for SA2394. In 4 out of 6 cases where I_NCX_ or I_NaL_ block was observed to be significant (IC50 < 30 µM), agreement classification improved. Results for SA5397 and SA7251 are illustrated in Figures 6B,C, and for other compounds, data can be found in Supplementary Table S1. Inclusion of I_NaL_ inhibition data significantly improved the match for SA5397 (Figure 6B), resolving prior discrepancies between simulated APD prolongation and experimental QT recordings. Simulations now align with experimental repolarization results (−6% at 10 µM) as no change in APD was observed, achieving quantitative agreement. Similarly, the inclusion of I_NCX_ inhibition improved agreement for SA7251 (Figure 6C). I_NCX_ inhibition reduced APD prolongation from +27.3% to +24.1% at 4.5 µM; experimental: +14%) and peak active tension (from −14.8% to +4.1%), aligning contractility predictions with experimental results (+23%).

The inclusion of NCX inhibition data demonstrated complex effects across the other 6 compounds, with examples improving one match (e.g., repolarisation) at the expense of the other. The specificity of IC50 data is critical when modelling NCX inhibition due to the model’s sensitivity to NCX conductance (as shown in the sensitivity analysis) and the bimodal nature of NCX current, which influences both contractility and repolarisation (Tomek et al., 2019).

Two unmatched compounds when using 6 channel data, dobutamine and SA7560 did not have specific ion channel data for key ionic channels which drive repolarisation and contractile changes. SA7560 only had specific data for I_NCX_ inhibition and all other inhibitions were estimated. Dobutamine only has contractility data for comparison, and no specific IC50 for I_CaL_ where dobutamine was shown to increase contractility in our *ex vivo* investigation; thus, it is expected that we would not observe a match.

## 4 Discussion

This study highlights the potential of NAMs, specifically human *in silico* drug trials, to replace conventional animal experiments for assessment of drug-induced effects on electrophysiology and contractility. Simulated action potential and active tension in human ventricular cardiomyocytes with four ionic current input data show predominantly quantitative agreement for 37 compounds across multiple concentrations with *ex vivo* rabbit whole-heart Langendorff drug screening. Consideration of additional input data on drug effects on sodium-calcium exchanger and late sodium current further improve the simulation-experimental match, particularly for contractility, as identified by a sensitivity analysis using populations of models to account for variability.

The main findings of this study are:

1. *In silico* predictions using the human electromechanical ventricular model (Margara et al., 2021) achieved qualitative matches for 92% of compounds, and quantitative contractility matches for 76%. For drug-induced repolarisation modulation, 85% and 64% of compounds showed qualitative and quantitative agreements respectively. Lower quantitative repolarisation matches often stemmed from strong hERG block, potentially reflecting species differences (Vandenberg et al., 2012), or lower-quality data.
2. Data quality was pivotal for accurate predictions. Compounds with complete dose-response curves and IC_50_ measurements yielded more quantitative agreements than those with incomplete or estimated data. For unmatched compounds, missing data (e.g., dobutamine lacking hCav1.2 IC_50_, or experimental APD data) was a key factor.
3. The sodium-calcium exchanger (NCX) emerged as an important factor for reproducing both repolarisation and contractility *in silico.* Including IC_50_ measurements for I_NCX_ improved agreement for several compounds with prior mismatches. Similarly, the inclusion of I_NaL_ inhibition data for the compound it was found to be significant led to a categorical improvement in agreement.

### 4.1 Human single cell *in silico* vs. whole-organ rabbit *ex vivo*

This study showcases the strong translatability of human-based simulations and *ex vivo* rabbit experiments in assessing electrophysiological and inotropic drug responses. Human-based simulations effectively predict clinical arrhythmogenesis and inotropic risk (Margara et al., 2022) offering a robust alternative to animal experiments while enhancing pharmaceutical discovery throughput (Musuamba et al., 2021). By capturing repolarisation and inotropic modulation concurrently, these simulations enable comprehensive evaluation of drugs with complex electrogenic and inotropic effects, such as Verapamil, Quinidine and Digoxin.

Sensitivity analysis identified the sodium-calcium exchanger current (I_NCX_) and the late sodium current (I_NaL_) as key drivers. Including hNCX1 and late NaV1.5 inhibition improved quantitative agreements for repolarisation and inotropic changes. High-quality IC_50_ data for pore block models yielded consistent quantitative matches, while mismatches correlated strongly with missing data, or estimated IC_50_ values. Dobutamine was the sole compound with no agreement, lacking experimental QT data, and hCav1.2 inhibition data. Simulations incorporating hNCX1 inhibition emphasized the need for high-quality inhibition data due to its dual-mode modulation of repolarisation and contractility, which affects both forward and reverse NCX currents.

### 4.2 Quantitative assessment between *in silico* and *ex vivo*

Rabbits are a widely used ventricular drug development model due to their similarly shaped action potential (Zicha et al., 2003), repolarisation reserve (Varró et al., 1993), and comparable cardiac tissue size relative to excitation wavelength (Panfilov, 2006). These characteristics make rabbits suitable for arrhythmic risk assessments (Blackwell et al., 2022) and inotropic evaluations as both species exhibit a positive force-frequency relationship (Milani-Nejad and Janssen, 2014).

Key disparities include higher and more variable Kv4.3 channel activity (Workman et al., 2012), increased SERCA2a flux (Su et al., 2003), faster heart rates, faster hERG channel deactivation and differences in I_Kr_ contributions to repolarisation (Ágoston et al., 2024). Furthermore, the input data used for ion channel drug block assume functional invariance of ion channel IC_50_ values between humans and rabbits (Tveito et al., 2020). Considering inter-species differences, our criteria of a similar trend within ±25% of the experimental value is a valid benchmark for quantitative agreement.

#### 4.2.1 Explaining disagreement between simulation and experiment for only one compound

Dobutamine was the sole compound showing no agreement, with specific IC_50_ data only for hERG and a non-specific IC_50_ for hCav1.2, hNav1.2 and hNCX1 channels. In healthy volunteers, studies on dobutamine have previously shown complex relations between plasma concentration, heart rate, blood pressure and contractility (Ahonen et al., 2008; Dubin and Mugno, 2024), as well as biphasic responses in stroke volume across multiple studies. These variable responses may contribute towards the inability to predict the dobutamine response using our input data. *Ex vivo* rabbit experiments showed a 17% increase in ventricular pressure at 0.1µM, consistent with findings from (Trovato et al., 2025), where dobutamine increased L-type calcium current by a factor of 1.22 at EC_50_. Our sensitivity analysis predicts that a similar gain-of-function increase of L-type calcium current conductivity would increase simulated peak active tension by ∼40%. Using the gain-of-function data in Trovato et al. (2025) to simulate dobutamine would have resulted in a quantitative agreement with our *ex vivo* rabbit data.

Compounds utilising NCX data, such as SA7251, showed increasing inotropic effects with concentration, while dobutamine, which does not inhibit NCX, highlights the need for gain-of-function data to improve agreement. Compounds matching only APD or inotropic modulation between simulation and experiment often lack specific data for hERG or hCav1.2 channels–the primary drivers of repolarisation and contractility. Accurate IC_50_ assessment from *in vitro* assays is critical for evaluating ion channel blockers *in silico* (Trovato et al., 2022).

### 4.3 Advantages of *in silico* approaches

High-throughput computational simulations offer a powerful alternative to animal experimentation by enabling the study of patient variability through virtual cohorts. Incorporating biological variability via a population of *in silico* ventricular cardiomyocyte models improves prediction reliability and robustness. For selected compounds, *in silico* trials were conducted on 166 human ventricular cardiomyocyte models with healthy phenotypes, incorporating variability through modified ionic current conductance. This study demonstrates how drug effects vary within a calibrated healthy adult human population, with increasing concentrations amplifying response diversity. Simulations with the population of models are consistent with the single cell simulations, reinforcing our confidence in the findings. The growing variability at higher drug concentrations highlights the necessity of considering patient differences when assessing therapeutic options. This approach can extend to specific cohorts, such as unhealthy individuals with myocardial infarction and heart failure (Zhou et al., 2024b; Zhou et al., 2024a; Qu et al., 2021) or hypertrophic cardiomyopathy (Doste et al., 2022; Passini et al., 2016) and include demographic factors including advanced age, sex or ethnicity (Holmes et al., 2025; Vicente et al., 2015). Expanding simulations to cover diverse drug conditions and cohort demographics enhances the detection of arrhythmogenic or inotropic risks.

Human-based simulations provide advantages over conventional *ex vivo* Langendorff experimentation. These non-animal methods align with the 3R’s principles—reduction, refinement, and replacement—advancing the goals of organizations like the UK government, European Commission, and World Health Organisation. NAMs streamline drug safety assessments, increase compound throughput, and accelerate development, overcoming experimental challenges such as drug solubility and enabling testing across diverse conditions (e.g., pacing frequencies, biological sex, pregnancy) and broader populations (Cui et al., 2018; Dönertaş et al., 2019; Kelleci Çelik and Karaduman, 2023). These *in silico* methodologies are cost-effective, utilizing standard computational equipment without the need for additional technologies, making them superior alternatives to expensive animal experiments.

### 4.4 Limitations and design choices

Comparative study between animal species and human has led to useful insights into key physiological mechanisms but using animal models quantitatively has been challenging due to inherent species differences (Tveito et al., 2020). This study provides a quantitative comparison between human *in silico* simulations and existing rabbit Langendorff drug screening data using high-throughput assessment often used in preclinical safety assessment.

The data generated by automated patch-clamp technique was taken from experiments performed at room temperature (21 °C), rather than physiological temperatures (33 °C or higher) due to increased success rate and stability in measured current block, and better agreement with published datasets (Trovato et al., 2022). It is known that electrophysiological parameters, including APD, are temperature dependent, which may have some impact on results; however, Syren et al. (2025) show a non-significant difference between APD measured at 28 °C and 37 °C, suggesting that APD may be relatively robust within this range.

We compared simulated APD_90_ outputs from fast single cell simulations to rabbit Langendorff QT intervals, isolating ionic-level drug effects. QT measurements inherently integrate transmural dispersion, lead placement and autonomic tone (Boukens et al., 2015) – factors excluded in our simulations which allow for a controlled comparison. Previous works demonstrate that changes in QTc track changes in APD90 closely (∆QTc/∆APD90 ∼ 0.8–1.1) (Boukens et al., 2015; Franz et al., 1987; Sedgwick et al., 1992). Similarly, we compared peak active tension with rabbit Langendorff ventricular pressure–focusing on myofilament response without confounding factors (preload, afterload and chamber geometry). Under isometric or tightly load-controlled conditions, changes in ventricular pressure are proportional to changes in active tension, scaled by geometry (Land et al., 2017; Walley, 2016), however spatial heterogeneity of ventricular wall stress is an important factor to consider and may play a role in only qualitative agreement for some compounds. The ±25% quantitative agreement margin sufficiently contains anticipated inter-beat and inter-observer variability under standard Langendorff perfusion (King et al., 2022; Louradour et al., 2023) and is comparable to other studies of this type (Passini et al., 2017; Trovato et al., 2025).

Our results demonstrate consistency between human-based *in silico* simulations and rabbit Langendorff experimental recordings across both repolarisation and contractility biomarkers. The simple Hill equation used in this study is sufficient for reproducing the drug effects *in silico* for most compounds; however, our simulations with dobutamine reveal that more complex models including gain-of-function would improve simulation accuracy. Furthermore, with additional experimental data, more complex compound-protein binding, for example, with Markov models, could reduce uncertainty in binding mechanisms (Lei et al., 2024) and may further improve the accurate simulation of drug effects *in silico* (Li et al., 2017). This however comes at an increase cost, with potential benefits that need to be evaluated further. The assumption of functional invariance on the ionic current response between species was used, as done in prior human-animal comparative studies (Passini et al., 2017; Tveito et al., 2020). Refining these assumptions with additional data could improve the *in silico* predictions of drug response (Joukar, 2021).

The sensitivity analysis presented I_NCX_ and I_NaL_ as two drivers of repolarization and contractile modulation which improved the matching of several compounds. Additionally, J_up_ (SR calcium uptake) was also significant in our sensitivity analyses, but their measurements were excluded as measurements of these fluxes would require more complex additional experimental procedures. Inclusion of additional data on these channels may further improve future *in silico* drug studies.

## Data Availability

The raw data supporting the conclusions of this article will be made available by the authors, without undue reservation.
